# Quantitative Proteomic Analysis of the Hfq-Regulon in *Sinorhizobium meliloti* 2011

**DOI:** 10.1371/journal.pone.0048494

**Published:** 2012-10-30

**Authors:** Patricio Sobrero, Jan-Philip Schlüter, Ulrike Lanner, Andreas Schlosser, Anke Becker, Claudio Valverde

**Affiliations:** 1 Laboratorio de Bioquímica, Microbiología e Interacciones Biológicas en el Suelo, Departamento de Ciencia y Tecnología, Universidad Nacional de Quilmes, Buenos Aires, Argentina; 2 Institute of Biology III, Faculty of Biology, University of Freiburg, Freiburg, Germany; 3 Core Facility Proteomics, Center for Biological Systems Analysis (ZBSA), Freiburg, Germany; Louisiana State University and A & M College, United States of America

## Abstract

Riboregulation stands for RNA-based control of gene expression. In bacteria, small non-coding RNAs (sRNAs) are a major class of riboregulatory elements, most of which act at the post-transcriptional level by base-pairing target mRNA genes. The RNA chaperone Hfq facilitates antisense interactions between target mRNAs and regulatory sRNAs, thus influencing mRNA stability and/or translation rate. In the α-proteobacterium *Sinorhizobium meliloti* strain 2011, the identification and detection of multiple sRNAs genes and the broadly pleitropic phenotype associated to the absence of a functional Hfq protein both support the existence of riboregulatory circuits controlling gene expression to ensure the fitness of this bacterium in both free living and symbiotic conditions. In order to identify target mRNAs subject to Hfq-dependent riboregulation, we have compared the proteome of an *hfq* mutant and the wild type *S. meliloti* by quantitative proteomics following protein labelling with ^15^N. Among 2139 univocally identified proteins, a total of 195 proteins showed a differential abundance between the Hfq mutant and the wild type strain; 65 proteins accumulated ≥2-fold whereas 130 were downregulated (≤0.5-fold) in the absence of Hfq. This profound proteomic impact implies a major role for Hfq on regulation of diverse physiological processes in *S. meliloti*, from transport of small molecules to homeostasis of iron and nitrogen. Changes in the cellular levels of proteins involved in transport of nucleotides, peptides and amino acids, and in iron homeostasis, were confirmed with phenotypic assays. These results represent the first quantitative proteomic analysis in *S. meliloti.* The comparative analysis of the *hfq* mutant proteome allowed identification of novel strongly Hfq-regulated genes in *S. meliloti*.

## Introduction

In bacteria, regulation of gene expression based on small non-coding RNA molecules (sRNAs) has emerged and consolidated as fundamental post-transcriptional checkpoints, usually involved in the response to environmental stress to maintain cell homeostasis [1]. In particular, trans-encoded sRNAs have the ability to establish imperfect complementary interactions with one or more mRNAs, thus affecting their translation rate and/or stability [2]. The activity of the RNA chaperone Hfq is often required to facilitate such interaction *in vivo* [3], [4]. Originally discovered as a host factor necessary for the replication of the coliphage Qβ [5], Hfq has been recognized as a key player in riboregulatory processes and a limiting factor for sRNA action [6]. The absence of this RNA-binding protein alters the steady concentration and/or stability of cellular sRNAs, leading to an abrogate response in the regulation of gene expression under certain conditions [7], [8].

The biological role of Hfq has been explored in diverse eubacterial taxons, namely α-proteobacteria [9], [10], β-proteobacteria [11], [12], γ-proteobacteria [13], [14] and Gram positives [15]. In the α-proteobacterium *Sinorhizobium meliloti* strain 2011, the absence of *hfq* promotes a pleiotropic phenotype [16], outlining the critical role of this protein in *S. meliloti* physiology. This microorganism is of particular interest because of its endosymbiotic association with the roots of legumes belonging to the *Medicago*-*Melilotus*-*Trigonella* tribe, in which these bacteria induce and colonize root nodule organs specifically devoted to nitrogen fixation [17], [18]. Some of the phenotypes observed in the *hfq* minus background of strain 2011 have been described in the related *S. meliloti* strain 1021, whose proteomic profile has been explored by 2D-PAGE [19]. Indeed, an *hfq* mutant serves to identify possible target mRNAs subject to direct or indirect Hfq-dependent riboregulatory processes [13], [20], [21].

Since its introduction in 2002 [22], the metabolic labelling strategy that uses stable isotopes in the growth medium has shown its potential for the determination of highly accurate quantitative proteomic profiles when coupled with high performance liquid chromatography and mass spectrometry [23], [24]. Still, this technical approach remains mostly unexplored in bacteria: only a few projects considered metabolic labelling with stable isotopes coupled with LC/MS [25], [26], [27] compared with the huge number of proteomic studies based on comparative 2D-PAGE profiles. In most cases, Hfq-dependent riboregulation is not an “all or nothing” process, and contributes to the fine tuning of target gene expression leading to modest regulatory factors. In this context, and due to its documented accuracy and sensitivity, high-throughput quantitative proteomic approaches that make use of metabolic labelling with stable isotopes, provide an excellent platform to quantify mild changes in gene expression at the protein level [24].

We here present the results of the first comparative quantitative proteomic analysis in the α-proteobacterial legume symbiont *S. meliloti* that made use of stable protein labelling with ^15^N. The study aimed to quantify the impact of knocking out the RNA chaperone Hfq on the proteome of *S. meliloti* strain 2011.

## Materials and Methods

### Bacterial Strains and Culture Conditions

In this work, *S. meliloti* 2011 [28] was used as the wild type strain. Strain 20PS01 is an isogenic mutant that bears an in-frame deletion of 63 bp within the central region of the *hfq* gene [16]. *S. meliloti* was cultured at 28°C in tryptone-yeast extract (TY; in g l^−1^: tryptone, 5; yeast extract, 3, CaCl_2_, 0.7) or in MOPS-buffered defined medium (MDM) [29]. When required, streptomycin was added to the growth medium at 400 µg/ml. For cytotoxicity growth assays, 5-fluorouracil (Sigma-Aldrich, USA), sodium glufosinate (BASTA, Bayer Crop Science, Argentina) or Bialaphos (Toku-E, USA) were added to the growth medium at the concentrations indicated in the text. In all cases, growth was monitored by measuring OD_600_ in cultures shaken at 120 rpm. For each test, three independent cultures were analysed and the experiments were repeated twice with similar results.

### ^15^N Isotopic Labelling of *S. meliloti* Cellular Proteins, and Preparation of Protein Samples

*S. meliloti* strains 2011 and 20PS01 were grown in 100 ml of MDM medium containing 0.1% w/v of ^14^NH_4_Cl, or ^15^NH_4_Cl, as the only nitrogen source. Cells were harvested at exponential phase by centrifugation at 5000×*g* for 10 min at 4°C. Proteins from harvested cells were separated into cytoplasmic, periplasmic and membrane fractions, as described in [30]. Cells were resuspended in 2 ml of TEX buffer [50 mM Tris/HCl (pH 8.0), 3 mM EDTA, 0.1% Triton X-100], incubated on ice for 45 min, centrifuged and washed with 2 ml of TEX buffer. The supernatants of both centrifugation steps were pooled, and kept as the ‘periplasmic fraction’. The remaining pellet was resuspended in 4 ml of 10 mM MgCl_2_, 50 mg ml^−1^ DNase A, 50 mg ml^−1^ RNase I, 20 mM Tris/HCl, pH 8.0, and incubated on ice for 30 min. After three passages through a French press at 20.000 psi, the resulting extract was freed of unbroken cells by centrifugation at 2000×*g* for 2 min. Broken cells were centrifuged at 160.000×*g* for 1.5 h at 4°C, yielding the supernatant as cytoplasmic fraction and the pellet as membrane fraction. Total protein content was quantified with the Bradford method [31]. The quality of protein fractions was verified by SDS-PAGE [32].

### Quantitative LC-MS Proteomic Analysis

#### Protein separation and digestion

The three fractions were reduced with DTT (50 mM, 10 min, 70°C), alkylated with iodoacetamide (120 mM, 20 min, room temperature) and separated by SDS-PAGE (Invitrogen, NuPAGE 4–12% Bis-Tris gels). The gels were stained with Simply Blue (Invitrogen). Each lane was cut into 24 bands. For in-gel digestion the excised gel bands were destained with 30% ACN, shrunk with 100% ACN, and dried in a Vacuum Concentrator (Concentrator 5301, Eppendorf, Hamburg, Germany). Digests with trypsin were performed overnight at 37°C in 0.05 M NH_4_HCO_3_ (pH 8.0). About 0.1 µg of protease was used for one gel band. Peptides were extracted from the gel slices with 5% formic acid.

#### LC-MS/MS analysis

LC-MS/MS analysis were performed on a Q-TOF mass spectrometer (Agilent 6520, Agilent Technologies) coupled to an 1200 Agilent nanoflow system via a HPLC-Chip cube ESI interface. Peptides were separated on a HPLC-chip with an analytical column of 75 µm i.d. and 150 mm length and a 40-nL trap column, both packed with Zorbax 300SB C-18 (5 µm particle size). Peptides were elutes with a linear acetonitrile gradient with 1%/min at flow rate of 300 nL/min (starting with 3% of acetonitrile).

The Q-TOF was operated in the 2 Ghz extended dynamic range mode. MS/MS analyses were performed using data-dependent acquisition mode. After a MS scan (2 spectra/s), a maximum of three peptides were selected for MS/MS (2 spectra/s). Singly charged precursor ions were excluded from selection. Internal calibration was applied using two reference masses.

#### Protein Identification

Mascot Distiller 2.3 was used for raw data processing and quantitation, essentially with standard settings for the Agilent Q-Tof. Mascot Server 2.3 was used for database searching with the following parameters: peptide mass tolerance: 20 ppm, MS/MS mass tolerance: 0.05 Da, enzyme: “trypsin” with 2 uncleaved sites allowed, variable modifications: Carbamidomethyl (C), Gln->pyroGlu (N-term. Q), oxidation (M). A custom made database containing all UniProt entries for the taxonomy *Sinorhizobium meliloti* (Swiss-Prot and TrEMBL) was used. All protein sequences of this target database were reversed, and the resulting decoy database was concatenated with the target database. On average the calculated protein false discovery rate (FDR) was 2%. The resulting lists of identified proteins were filtered by excluding all protein identifications with only one peptide.

#### Protein Quantitation

The individual L/H ratios for all peptides and the L/H ratios for all proteins have been calculated using Mascot Distiller Quantitation Toolbox (Matrix Science, London, UK). 98 atom % ^15^N incorporation was used to calculate the theoretical isotopic patterns of the heavy peptides. Only peptides with a Mascot peptide score equal or higher as the Mascot homology threshold have been used for quantitation. Proteins with less than 2 (or 3) peptides were excluded from quantitation. Mass time matches have been allowed, so that the identification of one peptide version (heavy or light) is sufficient for L/H ratio calculation. In addition, the following quality filters have been applied for excluding low quality data from quantitation: correlation threshold (matched rho) 0.7 and standard error threshold (elution profile correlation threshold) 0.1. Protein L/H ratios have been calculated as the median of the corresponding peptide ratios.

### Glutamine Synthetase Activity

Total GS activity was measured by the incorporation of hydroxylamine to γ-glutamyl hydroxamate from *S. meliloti* cell extract as described in [33], [34]. The GS unit was defined as the amount of enzyme that catalyses the production of 1 µmol γ-glutamyl hydroxamate per min. The cellular protein content was estimated with the Bradford Method [31].

### Siderophore Production

Siderophore production was measured in the supernatant of conditioned cultures in MDM medium with different iron supply, colorimetrically with the CAS method [35].

### Intracellular Iron Content

Cellular iron content was estimated with the commercial Liquid Fer-color AA kit (Wiener lab, Argentina). An equivalent of 20 OD_600_ of *S. meliloti* cells were harvested at exponential phase from cultures grown in MDM medium. Cell pellets were resuspended in 1 ml of saline solution (NaCl 0.85% w/v) and lysed by sonication. 100 µl of the cell extracts were used to estimate total protein content with the Bradford method, and 200 µl of the clarified supernatants were used for iron colorimetry.

## Results and Discussion

### The Absence of *hfq* Promotes Profound Changes in the *S. meliloti* Proteome

**Figure 1:**
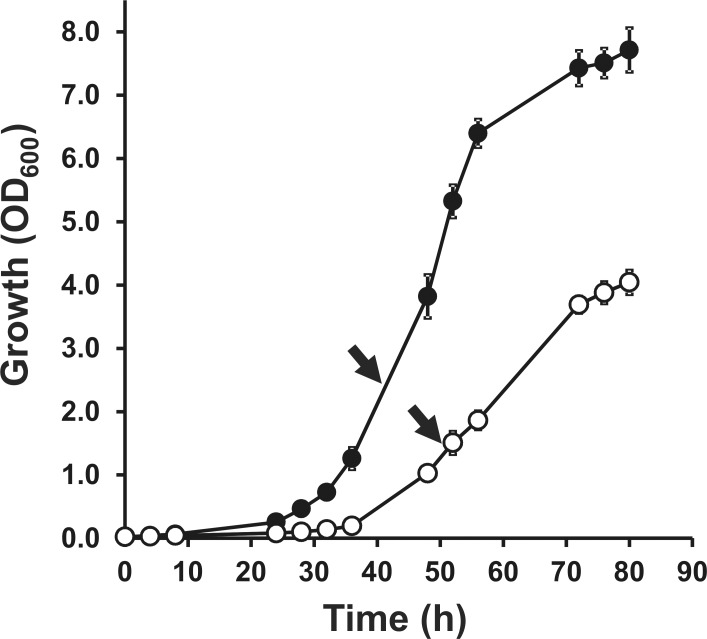
The absence of Hfq results in deficient growth in MDM defined medium. (•), wild type strain 2011; (○), Δ*hfq* mutant strain 20PS01. Each curve represents the average from three different cultures ± SD. The experiment was repeated twice, with essentially the same results. Arrows point to the growth stage in which cells were harvested for comparative quantitative analysis of ^15^N-labelled proteins.

In this study, we aimed to quantitatively characterize changes in the LC/MS protein profile of an *hfq* mutant derived from *S. meliloti* strain 2011 [16], after metabolic labelling of cellular proteins with stable ^15^N. In strain 2011, the absence of *hfq* generates a pleiotropic phenotype [16], which could be a consequence of a major proteome remodelling. This was evident as a growth deficiency in the defined MDM medium (Figure 1). For this reason, appropriate cell densities were chosen to ensure that cultures of wild type and *hfq* mutant cells were at a comparable growth phase (Figure 1). The quantitative proteomic analysis was carried out on protein extracts from exponential phase cultures growing in MDM medium containing ^15^NH_4_Cl as the only source of N. The efficiency of ^15^N incorporation was examined in the wild type strain 2011. After 6 generations in MDM medium with 0.1% (w/v) ^15^NH_4_Cl all identified proteins showed an incorporation of 98% ^15^N.

**Figure 2:**
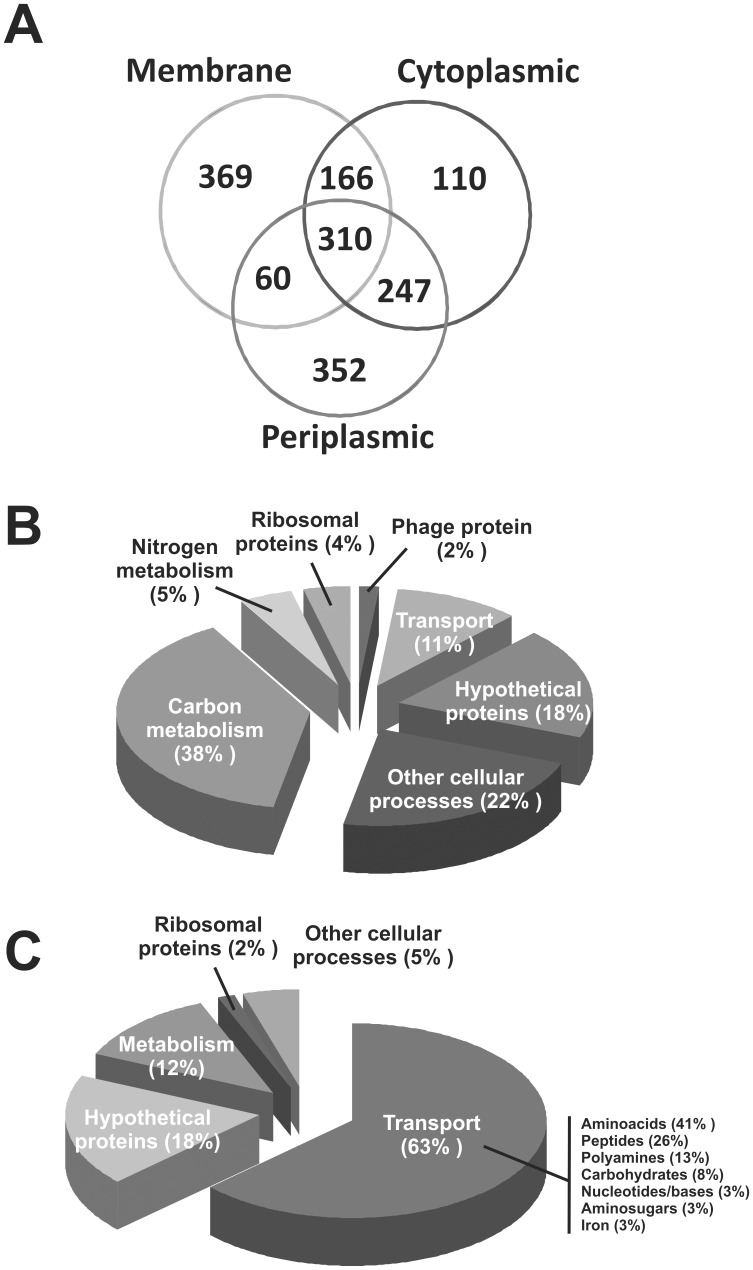
Summary of the quantitative LC/MS proteomic analysis of ^15^N labelled proteins. A) Representative Venn diagram of identified and quantified unique proteins in each of the subcellular fractions. B) Functional distribution of positively Hfq-regulated proteins. C) Functional distribution of negatively Hfq-regulated proteins.

In order to maximize the chances to identify regulated proteins, we carried out a subcellular protein fractionation, resulting in three different comparative protein profiles: cytoplasmic-, membrane- and periplasm-enriched fractions [30]. We could identify a total of 2139 unique proteins among the three fractions, which represented 34% of all the protein coding genes of *S. meliloti* 1021 genome [36]. In the cytoplasmic-enriched fraction, 833 proteins were quantified out of 1183 identified proteins. In the membrane-enriched fraction, 932 proteins were quantified out of 1276 identified proteins. Finally, in the periplasm-enriched fraction, 969 unique proteins were quantified out of 1258 identified proteins. Three biological replicates were analysed. In two of these replicates, the wild type strain *S. meliloti* 2011 was grown in the heavy nitrogen source, whereas the *hfq* mutant strain was grown in ^15^N in the third replicate. The high degree of correlation in the relative abundance of the quantified proteins discards any effect of the heavy nitrogen source on the proteomic profile. An important number of unique proteins was identified and quantified in only one fraction, supporting the subcellular fractionation strategy (Figure 2).

The relative abundance of each protein was expressed as average L/H ratios, which correspond to the relative abundance of a unique protein in the Δ*hfq* mutant strain 20PS01 (L) over that of the wild type strain 2011 (H). Thus, the quantitative proteomic analysis of ^15^N-labelled proteins allowed us to classify quantified proteins in three different groups: 1) proteins positively controlled by Hfq (those with L/H ratios <0.5); 2) proteins negatively regulated by Hfq (those with L/H ratios >2); 3) proteins whose relative abundance remained stable in the absence of Hfq (0.5< L/H <2) (Figure S1). The total number of proteins comprised into groups 1 and 2, raised to 195 unique polypeptides (Tables S1 and S2). Thus, 3% of the *S. meliloti* 2011 protein coding potential was detected as, directly or indirectly, controlled by Hfq in exponential growth phase in MDM medium. This figure most probably underestimates the real magnitude of the Hfq regulon in *S. meliloti*, as a large proportion of the encoded proteins were either expressed at levels below the detection limit or not expressed under the tested experimental condition. Previous studies on the impact of *hfq* deletion on global gene expression in *S. meliloti* revealed that, depending on the strain and growing condition, from 2.7% to 7% of the transcriptome [37], [38], and from 0.5% to 0.9% of the proteome [38], [39] were misregulated in the absence of Hfq. Thus, the results of our quantitative proteomic comparative study markedly enlarged the Hfq regulon of *S. meliloti*.

Importantly, the *hfq* in-frame internal deletion carried by strain 20PS01 was confirmed since the Hfq cellular levels were dramatically reduced (L/H = 0.05±0.01 in the membrane fraction and an average of 0.07 in two replicates of the cytoplasmic fraction). As for *E. coli*, in which a relevant fraction of the Hfq pool is closely associated to the cell membrane [40], the presence of Hfq in the membrane-enriched fraction may be partly due to its participation in riboregulatory mechanisms to control expression of the large set of membrane and secreted proteins of *S. meliloti* [41]. The level of the immediately downstream encoded gene product HflX was barely affected (L/H = 0.72) by the *hfq* deletion. Nevertheless, HflX could only be identified and quantified in one replicate of the membrane fraction. Even though, we can safely assume that the *hfq* mutation is not polar. Thus, it is expected that most, if not all, riboregulatory processes that require Hfq will be affected in the *hfq* mutant 20PS01.

### Proteins Positively Regulated by Hfq

In this section, we focus on unique proteins with L/H ratios <0.5 (Table S1). The majority of the positively controlled proteins (74%) are expressed from the chromosome of *S. meliloti*. In terms of biological functions, 38% are involved in carbon metabolism, such as alcohol dehydrogenase (SMa1296, L/H = 0.46±0.15 in the cytoplasmic fraction and 0.32±0.12 in the periplasm) or *smoS*, a putative manitol 2-dehydrogenase (SMc04093, L/H = 0.42±0.16 in the cytoplasmic fraction). Hypothetical proteins represented 18% of the proteins downregulated in the *hfq* mutant (Figure 2). In comparison with two recent studies that characterized the *S. meliloti* proteome in the absence of *hfq* by 2D-PAGE in strains 2011 [38] and 1021 [39], our quantitative proteomic analysis revealed 120 unique proteins with differential accumulation, which were not previously identified. The overlap of positively Hfq-controlled proteins was limited to only 2 proteins with Torres Quesada *et al.* (SMb20895, SMc04093) [38] and 8 proteins with Barra-Bily *et al.* (SMb21549, SMc01834, SMc02344, SMc02501, SMc02514, SMc02692, SMc02788 y SMc03157) [39].

A remarkable finding was that of the phage protein p077, as one of the least abundant protein in the *hfq* mutant (Table S1). p077 has been reported as a putative recombinase of the lysogenic phage 16-3 (UniprotKB RM163_077) [42]. More than 2% of *S. meliloti* 1021 genome represents mobile elements, such as phages or insertion sequences [36]. However, neither p077 nor other phage genes have been identified in the genome sequence of *S. meliloti* strain 1021 [36]. So, this mobile element could be specific of *S. meliloti* 2011.

The cobalamine-dependent ribonucleotide reductase NrdJ (SMc01237) was another strongly downregulated protein (L/H = 0.09, an average of the quantification in two biological replicates of the cytoplasmic fraction. Moreover, CobW (SMc04304), directly involved in the biosynthesis of cobalamine [43], also showed a reduced accumulation in the *hfq* mutant (L/H = 0.23, average from two replicates of the membrane fraction). As the biosynthesis of cobalamine is essential for the symbiosis between *S. meliloti* 1021 and *Medicago sativa* [44], [45], the observed depression of the cobalamine metabolism does not seem to block the symbiotic competence of the *hfq* mutant [16]. Interestingly, the corresponding transcripts of both detected proteins appeared downregulated only 2-fold in an *hfq* mutant [37], suggesting a major translational regulation by Hfq of *nrdJ* and *cobW* expression.

The SMc00986 protein was quantified among the strongest downregulated proteins, comparable to Hfq itself (Table S1), although its transcript showed a modest dependence on Hfq [37]. SMc00986 encodes a hypothetical protein with six DUF2117 domains, which are highly conserved domains in eubacterial proteins of unknown function. Interestingly, SMc00986 was found to be controlled by the iron master regulator RirA [46], as well as by CbrA [47] (a regulator of several envelope proteins in *S. meliloti*), and to be accumulated in the absence of the outer membrane protein TolC [48].

Structural proteins of the *S. meliloti* flagella were moderately repressed in the *hfq* mutant background. In the insoluble fraction enriched in membrane associated proteins, the relative abundance of FlaA, FlaB, FlaC and FlaD reached *ca*. 0.6 (L/H = 0.65±0.21, 0.60±0.28, 0.55±0.35 and 0.64±0.23, respectively). In the periplasmic fraction, the L/H ratio of FlaC was 0.93±0.4, whereas FlaA, FlaB and FlaD showed averages near 0.65 in two replicates of this fraction. In agreement with our observations, flagellar proteins were not detected as regulated by Hfq in previous proteomic studies [38], [39], and the corresponding transcripts were detected as barely downregulated in one of the two transcriptomic comparative analyses of *hfq* mutants [37]. These results suggest a modest contribution of Hfq in the control of flagellar structural genes. In turn, the reduced motility of *hfq* mutant cells derived from both *S. meliloti* 1021 and 2011 strains [16], [37] could not be attributed to major changes in the relative abundance of these proteins.

### Proteins Negatively Regulated by Hfq

Proteins whose L/H ratios resulted ≥2 were identified as negatively controlled by Hfq. In this group, 71% of the upregulated proteins derived from the *S. meliloti* chromosome (Figure 2), being 63% of them involved in transport of small molecules, notably L-amino acids or peptides (Figure 2). Among 65 quantified proteins with differential overexpression, 52 hits were only identified in this work (Table S2). Only a few upregulated proteins matched those identified by 2D-PAGE in previous studies: 6 proteins shared with Barra-Bily *et al*. [39] (SMc00140, SMc00242, SMc00770, SMc00784, SMc02884 and SMc03786), 3 proteins shared with Torres-Quesada *et al*. [38] (SMc02121, SMc02171, SMc02259), and 4 proteins that were common to all three proteomic studies (SMc00786, SMc01525, SMc01946, SMc02118). Thus, our quantitative proteomic analysis confirmed those hits and significantly expanded the list of candidate genes possibly subject to Hfq-dependent negative riboregulation.

As observed for proteins positively regulated by Hfq, the degree of overlap among proteomic studies is markedly low. Possible reasons for such apparent lack of consistency include differences in growth conditions (complex TY medium [38], defined GAS medium [39], or defined MDM medium in this study), growth phase of sampled cells (exponential [38], [39] or stationary [39]), strain (1021 [39] or 2011 [38]), mutant construction (*lacZ*::*accC1* insertion [39], plasmid insertion [38], or internal deletion [16]), and finally, the resolution power of the analytical technique (2D-SDS-PAGE followed by MS identification [38], [39], or LC-MS/MS analysis of ^15^N-labeled proteins in this study). However, the number of misregulated proteins detected in our work shows a greater degree of overlap with misregulated transcripts detected in microarray-based analyses of *hfq* mutants: 37 out of 130 polypeptides positively controlled by Hfq (Table S1) were reported as downregulated transcripts in *hfq* mutants in previous studies, whereas 26 out of 65 negatively controlled polypeptides were reported as upregulated transcripts in *hfq* mutants [37], [38]. Such correlations suggest that at least part of the Hfq-controlled genes is subject to direct riboregulatory mechanisms coupled to changes in mRNA stability, as reported for certain *E. coli* sRNAs [7], [49], [50]. By contrast, the level of mRNAs like SMc00986, SMc01237 (Table S1), SMc00786 and SMc02884 (Table S2) was proportionally less influenced by Hfq (1.8, 2.5, 1.5 and 1.6-fold, respectively) [37] than the corresponding proteins in our study (11.1, 20.0, 11.2 and 9.7-fold, respectively), indicating that the expression of these genes is mainly subject to translational Hfq-dependent regulation with less or no significant impact on mRNA stability.

It cannot be discarded that some of the observed changes in the *hfq* mutant could be also due to transcriptional control and the affected genes would thus represent indirect targets. The number of misregulated transcriptional regulatory proteins identified in the *hfq* mutant was very low, probably due to their typical low cellular concentration (Tables S1 and S2). However, for the two misregulated transcriptional regulators detected in our study (AniA and FrcR), we found that also one of the proteins under their control were co-regulated: AniA (SMc03880) and PhbB (SMc03879) [51] were upregulated *ca*. 2.8-fold in the *hfq* mutant (Table S2), whereas FrcR (SMc02172) and FrcA (SMc02169) [52] were downregulated *ca*. 3-fold in the Δ*hfq* strain (Table S1). Thus, part of the *S. meliloti* Hfq regulon may be a consequence of direct Hfq-dependent riboregulation of mRNAs encoding transcriptional regulators, resulting in indirect control on the subrogated mRNAs.

### Hfq Controls Uptake of Nitrogenous Compounds and N Metabolism

Several components of ABC transport systems possibly involved in uptake of oligopeptides were accumulated in the *hfq* mutant (Table S2): DppA1 (SMc00786), DppA2 (SMc01525), DppD2 (SMc01528) and DppF2 (SMc01529) are part of dipeptide transporters [53]; OppA (SMb21196) has been identified as a periplasmic binding component of an ABC transport system for di- and tripeptides [53]; all proteins encoded by the *aap* operon (*aapJQMP*; SMc02121, SMc02120, SMc02119, SMc02118), constitute a high affinity transport system for L-amino acids [41]; and several genes of the *liv* operon (*livHMGFK*; SMc01946, SMc01948, SMc01949, SMc01950, SMc01951) [41], are involved in the high affinity transport of leucine, valine and isoleucine. Several of the proteins encoded by these operons related to uptake of amino acid and small peptides were also previously reported to be negatively controlled by Hfq at either the protein or mRNA level [37], [38], [39] (Table S2), confirming the strong influence of Hfq on the flow of amino acids in *S. meliloti*. These findings point to the existence of Hfq-dependent sRNAs in *S. meliloti* targeting the mRNAs encoding those transporter proteins. sRNAs that control multiple transport systems have been identified in *Salmonella enterica* and *E. coli* (GcvB and RybB) [54], [55] and in the α-proteobacteria *Agrobacterium tumefaciens* (AbcR1) [56] and *Brucella abortus* (AbcR1 and AbcR2) [57]. GcvB and RybB homolog genes seem to be confined to Enterobacteriaceae (Rfam [58]), whereas AbcR1 and AbcR2 homologs have been identified in *S. meliloti* as the non-coding RNA transcripts SmrC15 and SmrC16 [59], [60], [61]. As suggested by computational predictions of mRNA targets (Text S1), SmrC15 and SmrC16 may fulfill the role of a multi-target sRNA controlling a number of proteins involved in small molecule transport. However, neither Dpp, Opp or Aap components have been identified *in silico* as SmrC15/C16 targets (Text S1). Conversely, the computational search of putative sRNAs encoded in *S. meliloti* intergenic regions which would be able to bind the mRNA leader of the *opp*, *liv*, *aap*, *dpp1* and *dpp2* operons did not identify the SmrC15/C16 sRNA genes nor revealed a single putative sRNA having all these operons as common targets (Text S1). Altogether, these computational analyses suggest that, most probably, several sRNAs (including SmrC15/C16) may be responsible for the strong Hfq-dependent riboregulation imposed to multiple oligopeptide and amino acid transporters in *S. meliloti*.

**Figure 3:**
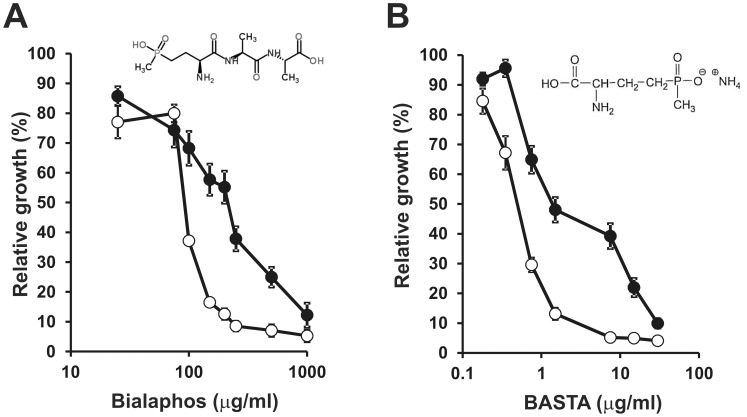
Bialaphos and sodium glufosinate sensitivity assay. In both cases, bacterial growth was estimated by OD_600_ measurements after 72 h in the presence of different concentrations of the toxic tripeptide Bialaphos (A) or sodium glufosinate (B). (•), wild type strain 2011; (○), Δ*hfq* mutant strain 20PS01. Data represent the growth measured for each *S. meliloti* strain in the presence of a given concentration of the toxic reagent relative to the growth in the absence of the chemical. Each bar shows average values for n = 3 replicate cultures ± SD. The experiment was repeated twice with similar results. The chemical structure of both chemicals is shown within each panel.

In order to get an *in vivo* correlation with the observed quantitative proteomic alterations on oligopeptide transporters, we tested the sensitivity of the *hfq* mutant strain 20PS01 to the antibacterial tripeptide Bialaphos. This herbicidal and antibiotic compound produced by some *Streptomyces* strains [62], presents two alanines and the glutamate toxic analogue phosphinotricine. Strain 20PS01 showed an increased sensitivity to Bialaphos (Figure 3); a concentration of 100 µg/ml inhibited the growth of the *hfq* mutant, whereas a concentration 2.5-fold higher was required to obtain the same degree of growth inhibition in the wild type strain (Figure 3). Most probably, the increased sensitivity to Bialaphos could be explained by the overexpression of oligopeptide transport systems such as Opp. In addition, the *hfq* mutant presented an increased sensitivity to ammonium glufosinate, an L-glutamate toxic analogue (Figure 3). As for Bialaphos, this sensitivity could be a direct effect of the accumulation of several proteins involved in the transport of L-amino acids, like the amino acid permease (Aap) transport system or other putative amino acid transporters (SMc02259, SMb20706). It is worth pointing out that the tight control of amino acid transporter expression by Hfq has also been recognized in the pea symbiont *Rhizobium leguminosarum* [63]. In this related α-proteobacterium, a number of spontaneous second site suppressor mutants were found to arise from a *gltB* mutant, which is unable to carry out *de novo* amino acids synthesis and shows reduced amino acid transport via Aap and Bra systems. Strikingly, 12 independent spontaneous mutants that regained growth on glutamate, had unique point mutations mapping within *hfq*, and showed strongly increased expression of several amino acid transport systems (Bra, Opp, App) [63]. Thus, negative control of amino acid and oligopeptide transport by Hfq seems to be a conserved feature among plant symbiotic rhizobia.

On the other hand, our analysis identified and quantified a group of transport proteins possibly involved in the uptake of polyamines, such as putrescine, agmatine and spermidine (SMa0392, SMb20284, SMc00770, SMc01652 and SMc01966) (Table S2). Among these, only PotF (SMc00770) has been previously identified as part of the Hfq regulon [39], although it was found to be repressed in an *hfq* mutant background in exponential phase in a defined medium [39]. Polyamines are key components involved in several physiological processes, such as transcription, mRNA stability, translation and oxidative stress resistance [64]. None of these proteins or putative polyamine transport systems has been studied in *S. meliloti* or other α-proteobacteria.

Altogether, our quantitative proteomic analysis revealed a major role of Hfq in the control of nitrogen metabolism of *S. meliloti* 2011 growing on ammonium as N source. Hence, changes in regulatory proteins that govern the nitrogen stress response could contribute to the observed changes in the *hfq* mutant background. The PII protein GlnB (SMc00947) was identified in our analysis but its level did not change significantly in the absence of *hfq*, whereas the PII protein GlnK and the regulators NtrB/C could not be identified in our study. On the other hand, the relative abundance of glutamine synthetase I (GlnAI or SMc00948) remained unaltered in the *hfq* mutant. Instead, GlnAI was upregulated ca. 3-fold in a *S. meliloti hfq* mutant in stationary phase cells [39]. Other enzymes with the same biological function, like GlnII (GlnT - SMc02613), SMc02352 or SMc01594, were not detected in our proteomic analysis of exponential phase cells. A probable glutamine synthetase (SMc00762) presented an L/H ratio of 0.42. Nevertheless, this deficiency would be complemented by GlnAI. The nitrogen assimilatory activity of glutamine synthetase (GS) was measured in order to get a picture of the nitrogen status in the absence of *hfq*. The GS activity in complex TY medium was not affected by the *hfq* deletion (Table 1). However, in the defined MDM medium containing NH_4_Cl (same growth conditions used for the quantitative proteomic analysis), the GS activity of the *hfq* mutant doubled that of the wild type strain (Table 1). Despite the fact that GlnAI levels remained constant, the higher GS activity could be due to a post-translational activation associated to changes in the intracellular level of GlnD, which in turn activates PII proteins and, finally, GS activity [65], [66], [67].

**Table 1:** Glutamine synthetase activity of an *S. meliloti* Δ*hfq* mutant growing in complex and defined media.

	Growth medium	
Strain (genotype)	TY	MDM-NH_4_^+^1% (w/v)
2011 (wild type)	2224.51±355.96 a	3221.69±574.29 a
20PS01 (Δ*hfq*)	2026.49±206.57 a	4741.83±974.29 b

Data correspond to average GS activity (Units/mg protein) of n = 3 replicate cultures ± SD. Different letters indicate statistically significant differences among strains based on pairwise comparisons (Student’s *t*-test, *P*<0.05).

### Hfq Control of Uracil/uridine Uptake

**Figure 4:**
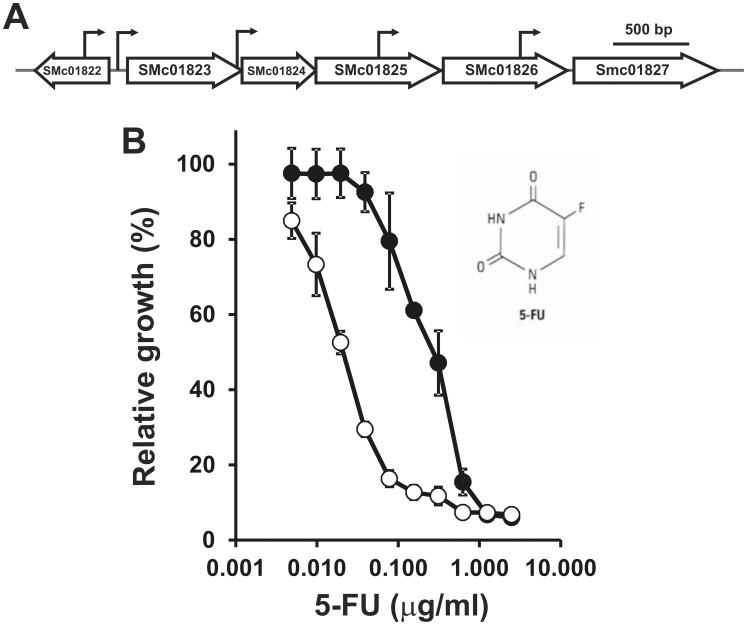
5-fluorouracil sensitivity assay. A) Schematic representation of the chromosomal *S. meliloti* 1021 locus encompassing the annotation SMc01827. The arrows indicate possible transcription start sites (J.-P. Schlüter & A. Becker, personal communication). B) Bacterial growth was estimated by OD_600_ measurements after 96 h in the presence of different concentrations of the uracil analogue. (•), wild type strain 2011; (○), Δ*hfq* mutant strain 20PS01. Values represent the relative growth of *S. meliloti* 2011 and 20PS01 in presence and absence of the toxic analogue. Each bar shows average values for n = 3 replicate cultures ± SD. The experiment was repeated twice with similar results. The chemical structure of the uracil analogue is shown.

A striking upregulation (>10-fold) was observed for the periplasmic substrate-binding component (SMc01827) from a putative ABC transport involved in the incorporation of uracil and/or uridine (Figure 4). SMc01827 showed the highest L/H ratios in the *hfq* mutant background (Table S2). In agreement with our finding, almost all members of the operon (Figure 4A) showed elevated mRNA levels in comparative microarray analyses of a *S. meliloti* 1021 and its isogenic Δ*hfq* mutant [37], [38]. These annotations, not yet functionally characterized in *S. meliloti*, seem to constitute the sole active transport system involved in the specific incorporation of uracil and/or uridine to be encoded in the *S. meliloti* genome. The accumulation of these proteins were verified *in vivo* by the incorporation of a cytotoxic uracil analogue 5-fluorouracil (5-FU) [68]. The *hfq* mutant presented a higher sensitivity to 5-FU (Figure 4). 5–10 ng per ml were sufficient to provoke growth inhibition of strain 20PS01, whereas in the wild type strain a comparable effect required 600 ng per ml of 5-FU (Figure 4). The accumulation of this ABC transport system would facilitate the uptake of 5-FU and reduce the tolerance to this agent. In order to discard an increased sensitivity of the *hfq* mutant to 5-FU rather than a more pronounced accumulation of the chemical, direct measurement of uracil uptake rate would be required. To our knowledge, we report for the first time evidences of Hfq-dependent regulation of uracil transport in bacteria.

### *hfq* Influences Iron Homeostasis in *S. meliloti* 2011

**Figure 5:**
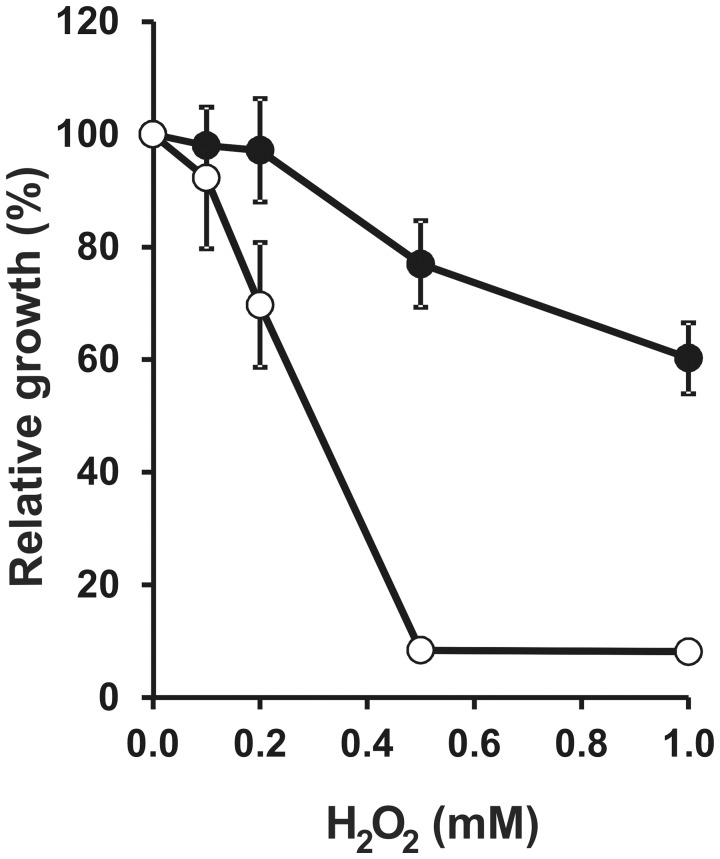
Hfq contributes to tolerance to oxidative stress. Bacterial growth in MDM was estimated by OD_600_ measurements after 72 h in the presence of increasing concentrations of H_2_O_2_. (•), wild type strain 2011; (○), Δ*hfq* mutant strain 20PS01. Values represent the relative growth of *S. meliloti* 2011 and 20PS01 in presence and absence of the oxidative stress agent. Each bar shows average values for n = 3 replicate cultures ± SD. The experiment was repeated twice with similar results.

Among the differentially expressed proteins identified in the *hfq* mutant, there was a set of proteins involved in the transport and storage of iron. Notably, the putative iron-storage bacterioferritin (Bfr or SMc03786) of *S. meliloti* 2011 accumulated in the Δ*hfq* strain (L/H = 2.41±0.23 in the cytoplasmic fraction, and L/H = 3.14±0.91 in the membrane fraction) (Table S2). In strain 1021, the opposite effect was reported: Bfr was downregulated in the *hfq* mutant [39]. The reasons for this discrepancy are unknown. Bacterioferritins have an important role in the homeostasis of iron [69], although its contribution to the control of cellular iron concentration has not been explored yet in *S. meliloti*. The intracellular iron content of the Δ*hfq* strain was 259.0±68.1 µmoles per mg of total protein, whereas the wild type strain contained 28.6±4.9 µmoles of iron per mg of total protein. This 9-fold increment in the cellular iron content could be explained by the higher availability of bacterioferritin detected in our quantitative analysis of the *S. meliloti* 2011 *hfq* mutant. Another evidence of an exacerbated iron accumulation response in the Δ*hfq* mutant was the upregulation of the putative Fe^+3^ transporters FbpA (SMc00784) and SMc01605 (Table S2). Higher than normal iron levels could lead to oxidative stress due to Fenton chemistry in the presence of reactive oxygen species like H_2_O_2_ [70]. The sensitivity of the *S. meliloti hfq* mutant was studied in the presence of H_2_O_2_, and we found an important growth inhibition in MDM medium under iron sufficient conditions (37 µM) (Figure 5). In line with the higher sensitivity to H_2_O_2_, the KatB peroxidase/catalase (SMa2379) was downregulated in the Δ*hfq* mutant (average L/H = 0.30, in two replicates of the membrane fraction) (Table S1). Similar results were reported for *S. meliloti* 1021 [39]. Other catalase/peroxidase proteins could not be identified in the protein profiles. Thus, the higher sensitivity to oxidative stress in the absence of *hfq* could be explained, at least partially, by the inability to activate expression of catalases involved in the detoxification process and, possibly, by a higher than normal level of free iron. As for the sensitivity to toxic analogs of amino acids and uracil (Figures 3 and 4), we cannot rule out that the higher sensitivity of the *hfq* mutant to H_2_O_2_ could be partially due to its slower growth rate (Figure 1). However, the observed phenotypes (Figures 3, 4, 5) are consistent with the quantitated changes in the cellular level of proteins directed involved in such phenotypes.

Considering that the iron concentration in the growth medium used for our proteomic analysis corresponded to an iron sufficient condition [46], the results led us to hypothesize that the genetic regulatory mechanisms operating on iron homeostasis genes are exacerbated in the absence of *hfq*. Under iron sufficient conditions, the Δ*hfq* mutant accumulates much more iron than the wild type strain. Thus, we studied the production of secreted iron-chelating compounds (siderophores) under different iron supplies in the wild type and Δ*hfq* mutant strains. As expected, both strains responded to lower iron availability by increasing the production of siderophores (Table 2). However, in the absence of *hfq*, more siderophores were produced in iron-limiting conditions and less iron-chelators were secreted in iron-sufficient conditions, with respect to the wild type strain (Table 2). Again, this pattern of siderophore response is consistent with a misregulated iron cellular level in the absence of Hfq.

**Table 2:** Hfq is required for normal siderophore production.

	Total iron concentration in the growth medium	
Strain (genotype)	0.37 µM	37 µM
2011 (wild type)	0.57±0.03 a	0.11±0.01 a
20PS01 (Δ*hfq*)	0.74±0.04 b	0.06±0.01 b

The results are presented as siderophore relative units measured by the CAS interference assay [35]. Values represent the average of three independent cultures ± SD. Different letters indicate statistically significant differences among strains based on pairwise comparisons (Student’s *t*-test, *P*<0.05.).

Altogether, these results reveal a critical role of *hfq* as a fine-tuning regulatory factor of iron homeostasis in *S. meliloti* 2011. This could be executed through a direct Hfq-dependent regulation of an iron-related global regulator like Fur, or, as in γ-proteobacteria, through Fur-regulated sRNAs of the RyhB family [71]. However, unlike γ-proteobacteria, the *S. meliloti* Fur homolog is dedicated to control Mn^+2^ uptake [72], whereas regulation of iron cellular levels relies on the transcriptional regulator RirA [46], and possibly on the yet uncharacterized iron response regulator Irr [73], [74]. Neither RirA nor Irr were detected as Hfq targets in this and previous studies (Tables S1 and S2) [37], [38], [39]. Yet, sRNAs under RirA or Irr control may contribute to iron homeostasis in *S. meliloti*. As there is no experimental evidence for the expression of RyhB-like sRNAs in *S. meliloti*, we scanned intergenic regions for the presence of putative RirA binding sites [74] located upstream annotated non-coding RNAs [75], following a similar strategy to that reported for identification of the *P. aeruginosa* Fur-regulated PrrF1 and PrrF2 sRNAs [76]. The search identified three annotated small transcripts just downstream putative RirA binding sites (Text S1). All three hits corresponded to transcripts detected by RNA pyrosequencing as cis-regulatory mRNA leaders linked to iron-metabolism genes (Text S1). Target mRNA searches for these annotated non-coding transcripts did not reveal obvious iron-related mRNAs (Text S1). Thus, the identification of bona-fide RyhB homolog(s) in *S. meliloti* remains an open task.

### Conclusions

The results described here represent the first quantitative proteomic approach in the α-proteobacterial legume symbiont *S. meliloti*, following stable protein labelling of cell cultures with ^15^N. The choice of the technical approach is relevant because: 1) post-transcriptional riboregulatory mechanisms may result in changes in protein levels without being reflected in changes in mRNA levels and thus escape transcriptomic profiling; 2) subtle Hfq-dependent control of gene expression requires sensitive techniques like quantitative proteomics of stable isotope labelled proteins. The outcome of the comparative protein profile in the absence of *hfq* significantly expands the repertoire of cellular processes influenced by the RNA chaperone Hfq in *S. meliloti*. Overall, nearly 200 polypeptides have been found to be misregulated in the Hfq mutant, including several polypeptides recently identified by 2D-PAGE [38], [39]. We reveal novel Hfq targets involved in the transport of small molecules, like peptides and uracil, and in iron homeostasis, and confirmed the associated phenotypes by *in vivo* assays. These results lead us to hypothesize that *S. meliloti* expresses sRNAs dedicated to regulate those cellular processes with the assistance of Hfq, like the γ-proteobacterial sRNAs GcvB and RybB, and the α-proteobacterial AbcR1 and AbcR2 sRNAs, that controls multiple ABC uptake systems [56], [57], [77], [78], and the regulatory sRNA RyhB that control iron homeostasis [7], [71]. The absence of *hfq* also affected the expression of genes encoding proteins involved in other cellular processes, like cell division and adaptation to heat shock. The corresponding genes or operons may be subject to either direct or indirect control by Hfq. A comparative expression analysis of translational reporter fusions to the putative target genes would help to confirm a direct control by Hfq.

## Supporting Information

Figure S1 **Comparative expression profiles of subcellular** ***S. meliloti*** **protein fractions.** Each point represents a single identified and quantified unique protein. The Y-axis represents the logarithm of the ratio of each polypeptide present in the Δ*hfq* mutant with respect to the wild type strain (log_10_ L/H), whereas the X-axis represents the logarithm of the number of unique peptides assigned to the same single polypeptide (log_10_ #). Proteins overexpressed in the Δ*hfq* mutant with have log_10_ L/H values >0.3, whereas repressed proteins have log_10_ L/H values <−0.3.
(TIF)

Table S1 **Proteins positively regulated by Hfq.** Proteins showing L/H ratios <0.5 for at least one subcellular fraction (C, cytoplasmic-enriched; M. membrane-enriched; P, periplasm-enriched) are listed by replicon (pSymA, pSymB or chromosome) according to their increasing annotation code. The number of biological replicates in which the polypeptide could be quantified is indicated between parentheses. For n = 3 biological replicates, average values are followed by its ± standard deviation.
(XLSX)

Table S2 **Proteins negatively regulated by Hfq.** Proteins showing L/H ratios >2.0 for at least one subcellular fraction (C, cytoplasmic-enriched; M. membrane-enriched; P, periplasm-enriched) are listed by replicon (pSymA, pSymB or chromosome) according to their increasing annotation code. The number of biological replicates in which the polypeptide could be quantified is indicated between parentheses. For n = 3 biological replicates, average values are followed by its ± standard deviation.
(XLSX)

Table S3 **Computational search of putative** ***S. meliloti*** **sRNAs with functional homology to enterobacterial GcvB and RyhB sRNAs.** This file compiles all results from the different approaches carried out to identify putative functional homologues of GcvB and RyhB sRNAs in *S. meliloti* 1021 genome. The procedure is fully described in the Text S1 file.
(XLS)

Text S1 **Computational search of putative** ***S. meliloti*** **sRNAs with functional homology to enterobacterial GcvB and RyhB sRNAs.** This file describes the different *in silico* approaches taken to identify sRNA genes in strain 1021 that may fulfill the same function as GcvB and RyhB sRNAs. Results from the different applied algorithms are shown in Table S3.
(DOC)

## References

[1] Storz G, Vogel J, Wassarman KM (2011) Regulation by Small RNAs in Bacteria: Expanding Frontiers. Mol Cell 43: 880–891. 21925377 10.1016/j.molcel.2011.08.022 PMC3176440

[2] Beisel CL, Storz G (2010) Base pairing small RNAs and their roles in global regulatory networks. FEMS Microbiol Rev 34: 866–882. 20662934 10.1111/j.1574-6976.2010.00241.x PMC2920360

[3] Sobrero P, Valverde C (2012) The bacterial protein Hfq: much more than a mere RNA-binding factor. Crit Rev Microbiol. 10.3109/1040841X.2012.664540 22435753

[4] Vogel J, Luisi BF (2011) Hfq and its constellation of RNA. Nat Rev Microbiol 9: 578–589. 21760622 10.1038/nrmicro2615 PMC4615618

[5] Franze de Fernandez MT, Eoyang L, August JT (1968) Factor fraction required for the synthesis of bacteriophage Qbeta-RNA. Nature 219: 588–590. 4874917 10.1038/219588a0

[6] Adamson DN, Lim HN (2011) Essential requirements for robust signaling in Hfq dependent small RNA networks. PLoS Comput Biol 7: e1002138. 21876666 10.1371/journal.pcbi.1002138 PMC3158044

[7] Masse E, Escorcia FE, Gottesman S (2003) Coupled degradation of a small regulatory RNA and its mRNA targets in Escherichia coli. Genes Dev 17: 2374–2383. 12975324 10.1101/gad.1127103 PMC218075

[8] Morita T, Maki K, Aiba H (2005) RNase E-based ribonucleoprotein complexes: mechanical basis of mRNA destabilization mediated by bacterial noncoding RNAs. Genes Dev 19: 2176–2186. 16166379 10.1101/gad.1330405 PMC1221888

[9] Robertson GT, Roop RM Jr (1999) The Brucella abortus host factor I (HF-I) protein contributes to stress resistance during stationary phase and is a major determinant of virulence in mice. Mol Microbiol 34: 690–700. 10564509 10.1046/j.1365-2958.1999.01629.x

[10] Kaminski PA, Desnoues N, Elmerich C (1994) The expression of nifA in Azorhizobium caulinodans requires a gene product homologous to Escherichia coli HF-I, an RNA-binding protein involved in the replication of phage Q beta RNA. Proc Natl Acad Sci U S A 91: 4663–4667. 8197116 10.1073/pnas.91.11.4663 PMC43848

[11] Sittka A, Pfeiffer V, Tedin K, Vogel J (2007) The RNA chaperone Hfq is essential for the virulence of Salmonella typhimurium. Mol Microbiol 63: 193–217. 17163975 10.1111/j.1365-2958.2006.05489.x PMC1810395

[12] Tsui HC, Leung HC, Winkler ME (1994) Characterization of broadly pleiotropic phenotypes caused by an hfq insertion mutation in Escherichia coli K-12. Mol Microbiol 13: 35–49. 7984093 10.1111/j.1365-2958.1994.tb00400.x

[13] Dietrich M, Munke R, Gottschald M, Ziska E, Boettcher JP, et al (2009) The effect of hfq on global gene expression and virulence in Neisseria gonorrhoeae. FEBS J 276: 5507–5520. 19691497 10.1111/j.1742-4658.2009.07234.x

[14] Sousa SA, Ramos CG, Moreira LM, Leitao JH (2010) The hfq gene is required for stress resistance and full virulence of Burkholderia cepacia to the nematode Caenorhabditis elegans. Microbiology 156: 896–908. 19942656 10.1099/mic.0.035139-0

[15] Christiansen JK, Larsen MH, Ingmer H, Sogaard-Andersen L, Kallipolitis BH (2004) The RNA-binding protein Hfq of Listeria monocytogenes: role in stress tolerance and virulence. J Bacteriol 186: 3355–3362. 15150220 10.1128/JB.186.11.3355-3362.2004 PMC415768

[16] Sobrero P, Valverde C (2011) Evidences of autoregulation of hfq expression in Sinorhizobium meliloti strain 2011. Arch Microbiol 193: 629–639. 21484295 10.1007/s00203-011-0701-1

[17] Gage DJ (2004) Infection and invasion of roots by symbiotic, nitrogen-fixing rhizobia during nodulation of temperate legumes. Microbiol Mol Biol Rev 68: 280–300. 15187185 10.1128/MMBR.68.2.280-300.2004 PMC419923

[18] Jones KM, Kobayashi H, Davies BW, Taga ME, Walker GC (2007) How rhizobial symbionts invade plants: the Sinorhizobium-Medicago model. Nat Rev Microbiol 5: 619–633. 17632573 10.1038/nrmicro1705 PMC2766523

[19] Barra-Bily L, Pandey SP, Trautwetter A, Blanco C, Walker GC (2010) The Sinorhizobium meliloti RNA chaperone Hfq mediates symbiosis of S. meliloti and alfalfa. J Bacteriol 192: 1710–1718. 20081033 10.1128/JB.01427-09 PMC2832522

[20] Zhang A, Wassarman KM, Rosenow C, Tjaden BC, Storz G, et al (2003) Global analysis of small RNA and mRNA targets of Hfq. Mol Microbiol 50: 1111–1124. 14622403 10.1046/j.1365-2958.2003.03734.x

[21] Ansong C, Yoon H, Porwollik S, Mottaz-Brewer H, Petritis BO, et al (2009) Global systems-level analysis of Hfq and SmpB deletion mutants in Salmonella: implications for virulence and global protein translation. PLoS One 4: e4809. 19277208 10.1371/journal.pone.0004809 PMC2652828

[22] Ong SE, Blagoev B, Kratchmarova I, Kristensen DB, Steen H, et al (2002) Stable isotope labeling by amino acids in cell culture, SILAC, as a simple and accurate approach to expression proteomics. Mol Cell Proteomics 1: 376–386. 12118079 10.1074/mcp.m200025-mcp200

[23] Emadali A, Gallagher-Gambarelli M (2009) [Quantitative proteomics by SILAC: practicalities and perspectives for an evolving approach]. Med Sci (Paris) 25: 835–842. 19849986 10.1051/medsci/20092510835

[24] Otto A, Bernhardt J, Hecker M, Becher D (2012) Global relative and absolute quantitation in microbial proteomics. Curr Opin Microbiol. 10.1016/j.mib.2012.02.005 22445110

[25] Soufi B, Kumar C, Gnad F, Mann M, Mijakovic I, et al (2010) Stable isotope labeling by amino acids in cell culture (SILAC) applied to quantitative proteomics of Bacillus subtilis. J Proteome Res 9: 3638–3646. 20509597 10.1021/pr100150w

[26] Auweter SD, Bhavsar AP, de Hoog CL, Li Y, Chan YA, et al (2011) Quantitative mass spectrometry catalogues Salmonella pathogenicity island-2 effectors and identifies their cognate host binding partners. J Biol Chem 286: 24023–24035. 21566117 10.1074/jbc.M111.224600 PMC3129184

[27] Vogels MW, van Balkom BW, Heck AJ, de Haan CA, Rottier PJ, et al (2011) Quantitative proteomic identification of host factors involved in the Salmonella typhimurium infection cycle. Proteomics 11: 4477–4491. 21919203 10.1002/pmic.201100224 PMC7167899

[28] Meade HM, Signer ER (1977) Genetic mapping of Rhizobium meliloti. Proc Natl Acad Sci U S A 74: 2076–2078. 266730 10.1073/pnas.74.5.2076 PMC431077

[29] McIntosh M, Krol E, Becker A (2008) Competitive and cooperative effects in quorum-sensing-regulated galactoglucan biosynthesis in Sinorhizobium meliloti. J Bacteriol 190: 5308–5317. 18515420 10.1128/JB.00063-08 PMC2493264

[30] Eggenhofer E, Haslbeck M, Scharf B (2004) MotE serves as a new chaperone specific for the periplasmic motility protein, MotC, in Sinorhizobium meliloti. Mol Microbiol 52: 701–712. 15101977 10.1111/j.1365-2958.2004.04022.x

[31] Bradford MM (1976) A rapid and sensitive method for the quantitation of microgram quantities of protein utilizing the principle of protein-dye binding. Anal Biochem 72: 248–254. 942051 10.1016/0003-2697(76)90527-3

[32] Laemmli UK (1970) Cleavage of structural proteins during the assembly of the head of bacteriophage T4. Nature 227: 680–685. 5432063 10.1038/227680a0

[33] Bender RA, Janssen KA, Resnick AD, Blumenberg M, Foor F, et al (1977) Biochemical parameters of glutamine synthetase from Klebsiella aerogenes. J Bacteriol 129: 1001–1009. 14104 10.1128/jb.129.2.1001-1009.1977 PMC235040

[34] Somerville JE, Kahn ML (1983) Cloning of the glutamine synthetase I gene from Rhizobium meliloti. J Bacteriol 156: 168–176. 6137474 10.1128/jb.156.1.168-176.1983 PMC215066

[35] Schwyn B, Neilands JB (1987) Universal chemical assay for the detection and determination of siderophores. Anal Biochem 160: 47–56. 2952030 10.1016/0003-2697(87)90612-9

[36] Galibert F, Finan TM, Long SR, Puhler A, Abola P, et al (2001) The composite genome of the legume symbiont Sinorhizobium meliloti. Science 293: 668–672. 11474104 10.1126/science.1060966

[37] Gao M, Barnett MJ, Long SR, Teplitski M (2010) Role of the Sinorhizobium meliloti global regulator Hfq in gene regulation and symbiosis. Mol Plant Microbe Interact 23: 355–365. 20192823 10.1094/MPMI-23-4-0355 PMC4827774

[38] Torres-Quesada O, Oruezabal RI, Peregrina A, Jofre E, Lloret J, et al (2010) The Sinorhizobium meliloti RNA chaperone Hfq influences central carbon metabolism and the symbiotic interaction with alfalfa. BMC Microbiol 10: 71. 20205931 10.1186/1471-2180-10-71 PMC2848018

[39] Barra-Bily L, Fontenelle C, Jan G, Flechard M, Trautwetter A, et al (2010) Proteomic alterations explain phenotypic changes in Sinorhizobium meliloti lacking the RNA chaperone Hfq. J Bacteriol 192: 1719–1729. 20081032 10.1128/JB.01429-09 PMC2832530

[40] Diestra E, Cayrol B, Arluison V, Risco C (2009) Cellular electron microscopy imaging reveals the localization of the Hfq protein close to the bacterial membrane. PLoS One 4: e8301. 20011543 10.1371/journal.pone.0008301 PMC2789413

[41] Mauchline TH, Fowler JE, East AK, Sartor AL, Zaheer R, et al (2006) Mapping the Sinorhizobium meliloti 1021 solute-binding protein-dependent transportome. Proc Natl Acad Sci U S A 103: 17933–17938. 17101990 10.1073/pnas.0606673103 PMC1635973

[42] Deak V, Lukacs R, Buzas Z, Palvolgyi A, Papp PP, et al (2010) Identification of tail genes in the temperate phage 16–3 of Sinorhizobium meliloti 41. J Bacteriol 192: 1617–1623. 20081029 10.1128/JB.01335-09 PMC2832519

[43] Rodionov DA, Vitreschak AG, Mironov AA, Gelfand MS (2003) Comparative genomics of the vitamin B12 metabolism and regulation in prokaryotes. J Biol Chem 278: 41148–41159. 12869542 10.1074/jbc.M305837200

[44] Campbell GR, Taga ME, Mistry K, Lloret J, Anderson PJ, et al (2006) Sinorhizobium meliloti bluB is necessary for production of 5,6-dimethylbenzimidazole, the lower ligand of B12. Proc Natl Acad Sci U S A 103: 4634–4639. 16537439 10.1073/pnas.0509384103 PMC1450223

[45] Taga ME, Walker GC (2010) Sinorhizobium meliloti requires a cobalamin-dependent ribonucleotide reductase for symbiosis with its plant host. Mol Plant Microbe Interact 23: 1643–1654. 20698752 10.1094/MPMI-07-10-0151 PMC2979309

[46] Chao TC, Buhrmester J, Hansmeier N, Puhler A, Weidner S (2005) Role of the regulatory gene rirA in the transcriptional response of Sinorhizobium meliloti to iron limitation. Appl Environ Microbiol 71: 5969–5982. 16204511 10.1128/AEM.71.10.5969-5982.2005 PMC1265945

[47] Gibson KE, Barnett MJ, Toman CJ, Long SR, Walker GC (2007) The symbiosis regulator CbrA modulates a complex regulatory network affecting the flagellar apparatus and cell envelope proteins. J Bacteriol 189: 3591–3602. 17237174 10.1128/JB.01834-06 PMC1855900

[48] Santos MR, Cosme AM, Becker JD, Medeiros JM, Mata MF, et al (2010) Absence of functional TolC protein causes increased stress response gene expression in Sinorhizobium meliloti. BMC Microbiol 10: 180. 20573193 10.1186/1471-2180-10-180 PMC2912261

[49] Ikeda Y, Yagi M, Morita T, Aiba H (2011) Hfq binding at RhlB-recognition region of RNase E is crucial for the rapid degradation of target mRNAs mediated by sRNAs in Escherichia coli. Mol Microbiol 79: 419–432. 21219461 10.1111/j.1365-2958.2010.07454.x

[50] Prevost K, Desnoyers G, Jacques JF, Lavoie F, Masse E (2011) Small RNA-induced mRNA degradation achieved through both translation block and activated cleavage. Genes Dev 25: 385–396. 21289064 10.1101/gad.2001711 PMC3042161

[51] Povolo S, Casella S (2000) A critical role for aniA in energy-carbon flux and symbiotic nitrogen fixation in Sinorhizobium meliloti. Arch Microbiol 174: 42–49. 10985741 10.1007/s002030000171

[52] Lambert A, Osteras M, Mandon K, Poggi MC, Le Rudulier D (2001) Fructose uptake in Sinorhizobium meliloti is mediated by a high-affinity ATP-binding cassette transport system. J Bacteriol 183: 4709–4717. 11466273 10.1128/JB.183.16.4709-4717.2001 PMC99524

[53] Nogales J, Munoz S, Olivares J, Sanjuan J (2009) Genetic characterization of oligopeptide uptake systems in Sinorhizobium meliloti. FEMS Microbiol Lett 293: 177–187. 19522956 10.1111/j.1574-6968.2009.01527.x

[54] Sharma CM, Darfeuille F, Plantinga TH, Vogel J (2007) A small RNA regulates multiple ABC transporter mRNAs by targeting C/A-rich elements inside and upstream of ribosome-binding sites. Genes Dev 21: 2804–2817. 17974919 10.1101/gad.447207 PMC2045133

[55] Urbanowski ML, Stauffer LT, Stauffer GV (2000) The gcvB gene encodes a small untranslated RNA involved in expression of the dipeptide and oligopeptide transport systems in Escherichia coli. Mol Microbiol 37: 856–868. 10972807 10.1046/j.1365-2958.2000.02051.x

[56] Wilms I, Voss B, Hess WR, Leichert LI, Narberhaus F (2011) Small RNA-mediated control of the Agrobacterium tumefaciens GABA binding protein. Mol Microbiol 80: 492–506. 21320185 10.1111/j.1365-2958.2011.07589.x

[57] Caswell CC, Gaines JM, Ciborowski P, Smith D, Borchers CH, et al (2012) Identification of two small regulatory RNAs linked to virulence in Brucella abortus 2308. Mol Microbiol 85: 345–360. 22690807 10.1111/j.1365-2958.2012.08117.x PMC3391331

[58] Gardner PP, Daub J, Tate J, Moore BL, Osuch IH, et al (2011) Rfam: Wikipedia, clans and the “decimal” release. Nucleic Acids Res 39: D141–145. 21062808 10.1093/nar/gkq1129 PMC3013711

[59] del Val C, Rivas E, Torres-Quesada O, Toro N, Jimenez-Zurdo JI (2007) Identification of differentially expressed small non-coding RNAs in the legume endosymbiont Sinorhizobium meliloti by comparative genomics. Mol Microbiol 66: 1080–1091. 17971083 10.1111/j.1365-2958.2007.05978.x PMC2780559

[60] del Val C, Romero-Zaliz R, Torres-Quesada O, Peregrina A, Toro N, et al (2012) A survey of sRNA families in alpha-proteobacteria. RNA Biol 9: 119–129. 22418845 10.4161/rna.18643 PMC3346310

[61] Valverde C, Livny J, Schluter JP, Reinkensmeier J, Becker A, et al (2008) Prediction of Sinorhizobium meliloti sRNA genes and experimental detection in strain 2011. BMC Genomics 9: 416. 18793445 10.1186/1471-2164-9-416 PMC2573895

[62] Schinko E, Schad K, Eys S, Keller U, Wohlleben W (2009) Phosphinothricin-tripeptide biosynthesis: an original version of bacterial secondary metabolism? Phytochemistry 70: 1787–1800. 19878959 10.1016/j.phytochem.2009.09.002

[63] Mulley G, White JP, Karunakaran R, Prell J, Bourdes A, et al (2011) Mutation of GOGAT prevents pea bacteroid formation and N2 fixation by globally downregulating transport of organic nitrogen sources. Mol Microbiol 80: 149–167. 21276099 10.1111/j.1365-2958.2011.07565.x

[64] Shah P, Swiatlo E (2008) A multifaceted role for polyamines in bacterial pathogens. Mol Microbiol 68: 4–16. 18405343 10.1111/j.1365-2958.2008.06126.x

[65] Yurgel SN, Kahn ML (2008) A mutant GlnD nitrogen sensor protein leads to a nitrogen-fixing but ineffective Sinorhizobium meliloti symbiosis with alfalfa. Proc Natl Acad Sci U S A 105: 18958–18963. 19020095 10.1073/pnas.0808048105 PMC2596199

[66] Yurgel SN, Rice J, Kahn M (2011) Nitrogen metabolism in S. meliloti?alfalfa symbiosis: Dissecting the role of GlnD and PII proteins. Mol Plant Microbe Interact. 10.1094/MPMI-09-11-0249 22074345

[67] Yurgel SN, Rice J, Mulder M, Kahn ML (2010) GlnB/GlnK PII proteins and regulation of the Sinorhizobium meliloti Rm1021 nitrogen stress response and symbiotic function. J Bacteriol 192: 2473–2481. 20304991 10.1128/JB.01657-09 PMC2863565

[68] Tomasz A, Borek E (1960) The Mechanism of Bacterial Fragility Produced by 5-Fluorouracil: The Accumulation of Cell Wall Precursors. Proc Natl Acad Sci U S A 46: 324–327. 16578486 10.1073/pnas.46.3.324 PMC222834

[69] Carrondo MA (2003) Ferritins, iron uptake and storage from the bacterioferritin viewpoint. EMBO J 22: 1959–1968. 12727864 10.1093/emboj/cdg215 PMC156087

[70] Cornelis P, Wei Q, Andrews SC, Vinckx T (2011) Iron homeostasis and management of oxidative stress response in bacteria. Metallomics 3: 540–549. 21566833 10.1039/c1mt00022e

[71] Salvail H, Masse E (2012) Regulating iron storage and metabolism with RNA: an overview of posttranscriptional controls of intracellular iron homeostasis. Wiley Interdiscip Rev RNA 3: 26–36. 21793218 10.1002/wrna.102

[72] Platero R, Peixoto L, O’Brian MR, Fabiano E (2004) Fur is involved in manganese-dependent regulation of mntA (sitA) expression in Sinorhizobium meliloti. Appl Environ Microbiol 70: 4349–4355. 15240318 10.1128/AEM.70.7.4349-4355.2004 PMC444773

[73] Johnston AW, Todd JD, Curson AR, Lei S, Nikolaidou-Katsaridou N, et al (2007) Living without Fur: the subtlety and complexity of iron-responsive gene regulation in the symbiotic bacterium Rhizobium and other alpha-proteobacteria. Biometals 20: 501–511. 17310401 10.1007/s10534-007-9085-8

[74] Rodionov DA, Gelfand MS, Todd JD, Curson AR, Johnston AW (2006) Computational reconstruction of iron- and manganese-responsive transcriptional networks in alpha-proteobacteria. PLoS Comput Biol 2: e163. 17173478 10.1371/journal.pcbi.0020163 PMC1698941

[75] Schluter JP, Reinkensmeier J, Daschkey S, Evguenieva-Hackenberg E, Janssen S, et al (2010) A genome-wide survey of sRNAs in the symbiotic nitrogen-fixing alpha-proteobacterium Sinorhizobium meliloti. BMC Genomics 11: 245. 20398411 10.1186/1471-2164-11-245 PMC2873474

[76] Wilderman PJ, Sowa NA, FitzGerald DJ, FitzGerald PC, Gottesman S, et al (2004) Identification of tandem duplicate regulatory small RNAs in Pseudomonas aeruginosa involved in iron homeostasis. Proc Natl Acad Sci U S A 101: 9792–9797. 15210934 10.1073/pnas.0403423101 PMC470753

[77] Sharma CM, Papenfort K, Pernitzsch SR, Mollenkopf HJ, Hinton JC, et al (2011) Pervasive post-transcriptional control of genes involved in amino acid metabolism by the Hfq-dependent GcvB small RNA. Mol Microbiol 81: 1144–1165. 21696468 10.1111/j.1365-2958.2011.07751.x

[78] Balbontin R, Fiorini F, Figueroa-Bossi N, Casadesus J, Bossi L (2011) Recognition of heptameric seed sequence underlies multi-target regulation by RybB small RNA in Salmonella enterica. Mol Microbiol 78: 380–394. 10.1111/j.1365-2958.2010.07342.x 20979336

