# Correction: Quantitative Proteomic Analysis of the Hfq-Regulon in *Sinorhizobium meliloti* 2011

**DOI:** 10.1371/journal.pone.0175832

**Published:** 2017-04-10

**Authors:** Patricio Sobrero, Jan-Philip Schlüter, Ulrike Lanner, Andreas Schlosser, Anke Becker, Claudio Valverde

There are errors in the sixth sentence of the *hfq* Influences Iron Homeostasis in *S*. *meliloti* 2011 section of the Results and Discussion. The correct sentence is: The intracellular iron content of the Δ*hfq* strain was 4.28±1.13 nmoles per mg of total protein, whereas the wild type strain contained 0.47±0.08 nmoles of iron per mg of total protein.
