# Supplementary figures and images for: Quantitative Proteomic Analysis of the Hfq-Regulon in Sinorhizobium meliloti 2011
Source: PLoS One. 2012 Oct 30;7(10):e48494. doi: 10.1371/journal.pone.0048494 (PMC3484140; doi:10.1371/journal.pone.0048494)

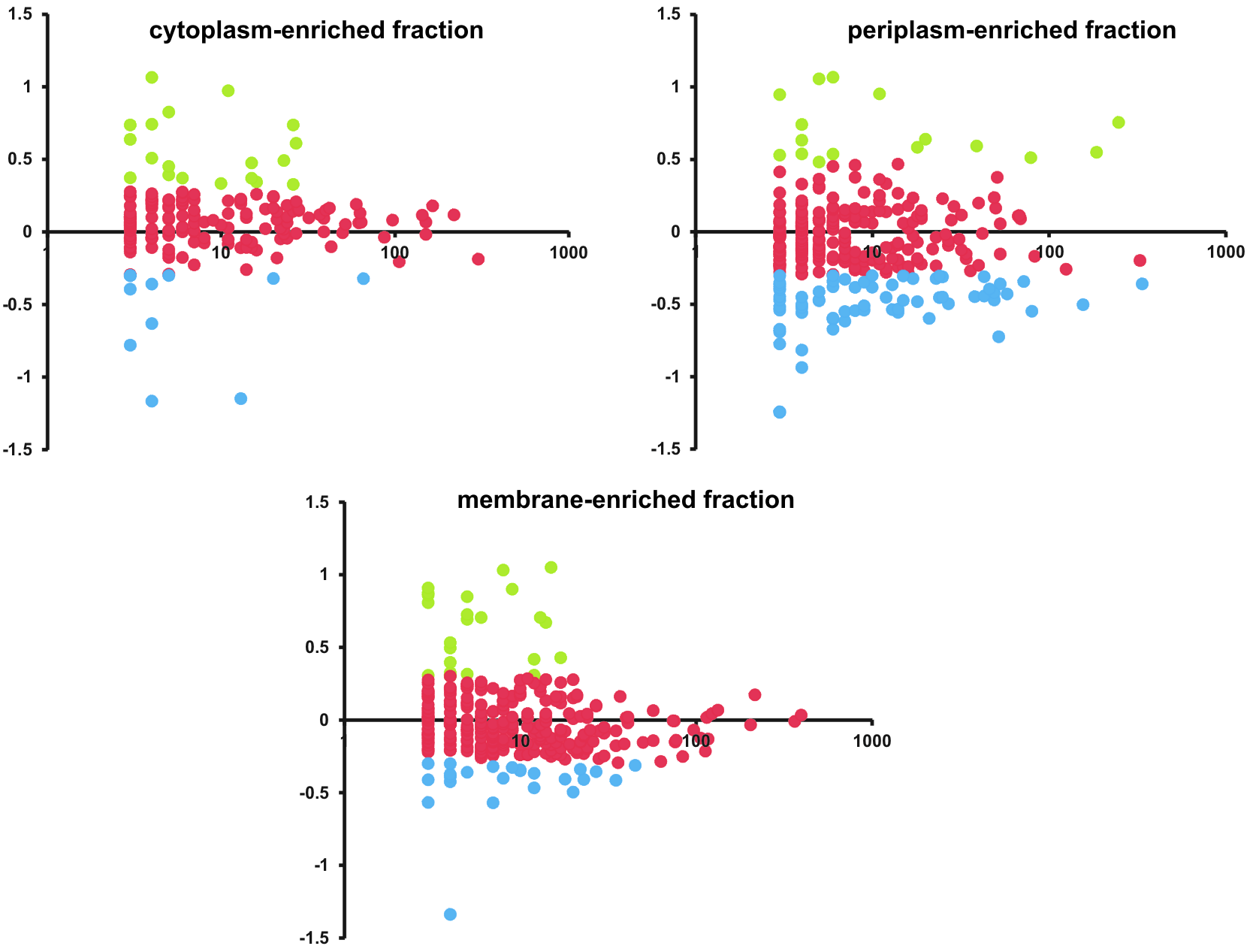

Supplement: Figure S1 — Comparative expression profiles of subcellular S. meliloti protein fractions. Each point represents a single identified and quantified unique protein. The Y-axis represents the logarithm of the ratio of each polypeptide present in the Δhfq mutant with respect to the wild type strain (log10 L/H), whereas the X-axis represents the logarithm of the number of unique peptides assigned to the same single polypeptide (log10 #). Proteins overexpressed in the Δhfq mutant with have log10 L/H values >0.3, whereas repressed proteins have log10 L/H values <−0.3. (TIF) [file pone.0048494.s001.tif]
