# Supplementary material for: Quantitative Proteomic Analysis of the Hfq-Regulon in Sinorhizobium meliloti 2011
Source: PLoS One. 2012 Oct 30;7(10):e48494. doi: 10.1371/journal.pone.0048494 (PMC3484140; doi:10.1371/journal.pone.0048494)
Supplement: Text S1 — Computational search of putative S. meliloti sRNAs with functional homology to enterobacterial GcvB and RyhB sRNAs. This file describes the different in silico approaches taken to identify sRNA genes in strain 1021 that may fulfill the same function as GcvB and RyhB sRNAs. Results from the different applied algorithms are shown in Table S3. (DOC) [file pone.0048494.s005.doc]

**Computational search of putative *S. meliloti* sRNAs with functional homology to enterobacterial GcvB and RyhB sRNAs.**

**1.** Is there any sRNA candidate in *S. meliloti* which resembles **GcvB** sRNA from *E.coli/Salmonella* or AbcR1 from *Agrobacterium*, which have been shown to directly repress expression of amino acid/peptide transporters?

In this work, several components of ABC transport systems possibly involved in uptake of oligopeptides were accumulated in the *hfq* mutant (Table S2): DppA1 (SMc00786), DppA2 (SMc01525), DppD2 (SMc01528) and DppF2 (SMc01529) are part of dipeptide transporters; OppA (SMb21196) has been identified as a periplasmic binding component of an ABC transport system for di- and tripeptides; all proteins encoded by the *aap* operon (*aapJQMP*; SMc02121, SMc02120, SMc02119, SMc02118), constitute a high affinity transport system for L-amino acids; and several genes of the *liv* operon (*livHMGFK*; SMc01946, SMc01948, SMc01949, SMc01950, SMc01951), are involved in the high affinity transport of leucine, valine and isoleucine.

In *E. coli* and *S. enterica*, the *trans*-encoded sRNA GcvB controls expression of multiple ABC transporters of di- and oligopeptides [1,2]. In the α-proteobacteria *Agrobacterium tumefaciens* and *Brucella abortus*, a couple of homolog sRNAs termed AbcR1 and AbcR2, are also involved in control of several transporter systems [3,4]. It was therefore hypothesized that *S. meliloti* expresses one or more sRNA genes that are functional homologs of GcvB, and/or AbcR1/R2. In order to search for putative GcvB-like sRNA genes, we carried out the following analyses:

1.
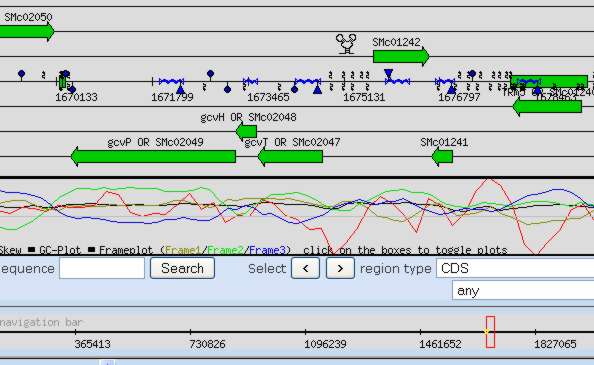
Direct search of a GcvB homolog in *S. meliloti*: the *E. coli* GcvB sequence was used as a query to look for homolog sequences in the genome of *S. meliloti* strain 1021 using BlastN with default parameters and E<10000. No hits were revealed. Next, we explored the Rfam database (http://rfam.sanger.ac.uk/browse) for annotated homologs of GcvB in α-proteobacteria; however, GcvB gene sequences seem to be confined to γ-proteobacteria. Finally, in *E. coli* and *Salmonella*, the GcvB sRNA gene is encoded in the intergenic region downstream *gcvA*, which encodes the transcriptional activator of the *gcvTHP* operon. The corresponding transcriptional regulator of the *S. meliloti* *gcvTHP* operon has not been identified, and there is no annotated putative transcriptional regulator flanking the *gcvTHP* operon in *S. meliloti* strain 1021. There is, however, an annotated *trans*-encoded sRNA gene within the *gcvTHP* – SMc01242 intergenic (IgR) region, which is divergently transcribed from the *gcv* operon. The sRNA gene is SmelC399 (see Figure) (https://gendb.cebitec.uni-bielefeld.de/cgi-bin/login.cgi?project=GenDB_S._meliloti_Public&login). In order to explore a possible functional relationship to GcvB, we carried out target mRNA prediction for SmelC399 using TargetRNA (http:// http://snowwhite.wellesley.edu/targetRNA/) and IntaRNA (http://rna.informatik.uni-freiburg.de:8080/IntaRNA.jsp), with default parameters (seed = 7 base pairs; mRNA pairing between positions -30 to +20, with respect to start codon). Among 100 predicted mRNA targets, only 4 were identified by both algorithms, and none of them corresponded to genes related to nitrogen uptake (see Table S3). Among the predicted mRNA targets identified by one of the two algorithms, there are 6 mRNAs related to N uptake (SMc04034, SMc04126, SMc00243, SMc01653, dppD1 and SMc03827); of these, the corresponding polypeptides of SMc00243, SMc01653 and DppD1, or members of their operons, were upregulated in the proteome of the *hfq* mutant (Table S2).

b. Are there functional homologs of the *Agrobacterium tumefaciens* and *Brucella abortus* AbcR1/AbcR2 sRNAs genes in *S. meliloti*?: In *Agrobacterium tumefaciens*, AbcR1 negatively controls expression of mRNAs encoding transporters (Atu2422, Atu1879 and *frcC*) [4] whereas the *Brucella abortus* sRNAs AbcR1 and AbcR2 negatively control the cellular level of several transported related to amino acid and polyamine transport [3]. AbcR1/R2 are members of the α-proteobacterial RNA family αr15, which includes the *S. meliloti* sRNA genes SmrC15 and SmrC16 [5]. Moreover, the physical location of the SmrC15/C16 genes is syntenic to that of *A. tumefaciens* AbcR1/R2 [4,6]. Based on these observations, we carried out prediction of mRNA targets for the *S. meliloti* sRNAs SmrC15/C16.

We used TargetRNA to identify *S. meliloti* 1021 mRNAs able to pair at ±50 bases around the start codon, with a seed region of 7, 9, or 11 bases (with or without G:U pairs) and IntaRNA with a seed region of 7 bases (default includes G:U pairing) [7]. Results are summarized in Table S3.

First, neither members of the *dpp*, *opp* nor *aap* operons were detected as putative targets of both sRNAs, using both algorithms. No common hits were revealed by TargetRNA and IntaRNA for SmrC15, whereas 6 mRNAs were detected by both algorithms using SmrC16 as a query, being both of them (SMb20284 and SMb20442) putative transporters of nitrogenated compounds.

Second, IntaRNA revealed a significant high proportion of different types of transporter mRNAs as putative hits for SmrC15 and SmrC16. For the three *S. meliloti* replicons, 35-45% of the top scoring hits for SmrC15 are involved in transport of small molecules (most of them not related to nitrogen), whereas 40-55% did so for SmrC16. These figures fell to 26% and 28%, respectively, for TargetRNA predictions.

Third, both searches, with their own criteria, identified a number of putative mRNA hits that are common to both sRNAs, suggesting the existence of certain degree of overlap among the regulons of both sRNA genes. For instance, 8 mRNAs were common to SmrC15 and SmrC16 for the TargetRNA output (including LivG (SMc01949), a member of *livHMGF* operon, that is strongly upregulated in the *hfq* mutant; Table S2), and 12 mRNAs were common to SmrC15 and SmrC16 within the top scored IntaRNA hits.

c. Is there any *S. meliloti* annotated sRNA that targets the Hfq-dependent ABC transporters Opp, Liv, Aap and Dpp?

In order to identify possible *S. meliloti* sRNA genes which may act as a global regulator of multiple ABC transport systems devoted to uptake of amino acids and small peptides, we carried out a reverse search using the TargetRNA tool to scan *S. meliloti* genomic IgRs for possible antisense pairing with the leader regions of the *opp*, *aap, liv* and *dpp* operons.

One difficulty with this approach is the lack of information about the transcriptional start site of each mRNA. We then delimited the search to ±50 bases around the start codon of the first gene of the operon (*appA*, *dppA1*, *livH*, *dppA2* and *oppA*), which indeed introduces another bias, because the sRNA regulation may not only be addressed to the first gene within an operon, or exclusively to the ribosome access region [8]. The minimal seed region was set up to 7 bases, including G:U pairings. The results are presented in Table S3.

First, there was no single common IgR identified as putative antisense sequence to all 5 queried target mRNA leaders. We found in turn, 4 IgRs that were common to only two of the target mRNAs. In three IgRs, there were annotated sRNA transcripts, but none of them overlapped the predicted pairing region for the corresponding IgR.

***Conclusions***

To summarize: a) direct search of sequence or structural homologs, nor of syntenic sRNA genes, allowed identification of a *S. meliloti* *gcvB*-like gene; b) computational evidences do not support the notion that the *S. meliloti* AbcR1/R2 sRNA sequence homologs, denoted as SmrC15 or SmrC16, are functional homologs of GcvB in that they have the potential to base pair simultaneously the mRNAs of different members of ABC transporters involved in amino acid and small peptide uptake (i.e., Dpp, Opp or Aap). However, SmrC15 and SmrC16 may fulfill the role of multi-target sRNAs controlling a number of other mRNAs encoding proteins involved in small molecule transport; c) the computational search of putative sRNAs encoded within *S. meliloti* intergenic regions which would be able to bind the mRNA leader of the *opp*, *liv*, *aap*, *dpp1* and *dpp2* operons, did not identify the SmrC15/C16 sRNA genes nor revealed a single putative sRNA having all these operons as common targets. Altogether, these computational analyses suggest that, most probably, several sRNAs may be responsible for the strong Hfq-dependent riboregulation imposed to multiple oligopeptide and amino acid transporters in *S. meliloti*.

**2.** Is there any potential homolog of **RyhB** sRNA in *S. meliloti*?

In *E. coli* and *Salmonella enterica*, the ferric uptake regulator protein Fur negatively controls transcription of the small regulatory RNA RyhB, which in turn negatively controls translation and mRNA stability of a number of mRNAs encoding proteins that store or make use of iron [9]. Unlike γ-proteobacteria, the *S. meliloti* Fur homolog is dedicated to control Mn^+2^ uptake [10], whereas regulation of iron cellular levels relies on the transcriptional regulator RirA [11], and possibly on the yet uncharacterized iron response regulator Irr [12]. Neither RirA nor Irr were detected as Hfq targets in this or previous studies (Tables S1 and S2). Yet, sRNAs under RirA or Irr control may contribute to iron homeostasis in *S. meliloti*.

It was therefore hypothesized that *S. meliloti* encodes one or more sRNA genes that are functional homologs of RyhB. In order to search for putative RyhB-like sRNA genes, we carried out the following analyses:

a. Direct search of an RyhB homolog in *S. meliloti*: the *E. coli* RyhB sequence was used as a query to look for homolog sequences in the genome of *S. meliloti* strain 1021 using BlastN with default parameters and E<10000. No hits were revealed. Next, we explored the Rfam database (http://rfam.sanger.ac.uk/browse) for annotated homologs of RyhB in α-proteobacteria; however, as for GcvB, RyhB gene sequences seem to be confined to γ-proteobacteria.

b. Is there any *S. meliloti* annotated sRNA bearing a putative RirA binding sequence overlapping its promoter?: based on the list of *S. meliloti* genes that were experimentally demonstrated to be controlled by RirA [11], a consensus matrix was built by multiple alignment of their promoter regions using MEME (http://meme.sdsc.edu/meme/cgi-bin/meme.cgi) and used to query the replicons of *S. meliloti* 1021 to search for putative RirA-binding sites in intergenic regions using MAST (http://meme.sdsc.edu/meme/cgi-bin/mast.cgi). The strategy was successful in that many of the previously reported RirA-controlled genes were identified as ORFs flanking IgRs with putative RirA-boxes. Next, we explored all the IgRs with predicted RirA-boxes (E<1) for the presence of sRNA transcripts [13] or sRNA predicted gene sequences [14].

Three IgRs were identified, one in the chromosome and two in the pSymA megaplasmid. The identified loci are:


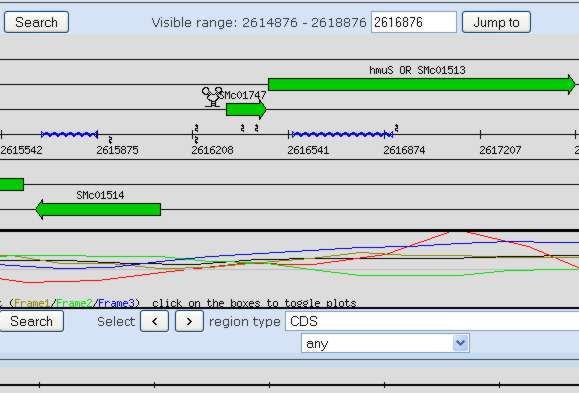
**>SmelC593**

2616210_2616344 (SMc01514_SMc01747)

CTGTCCGCGTCGGTCCATTGGGTCCCCGCACGCGTTGCTTTTAAGTTGACCGCAACATTCCGGTATTGCGTCCTTAAAGAAATATGAGTAATGAAGTCAAGATACTAAATGGAATGGGCGGGACCAATGGATGACGCGCTCGTCTCGTCTCCAACTCGCGAATTGGCAAGACCGTGACACCGAACGACACCGATAATCCACGATCCCCGCAAACCGTGGCGCCGCTGAACCGGCC

RirA boxes

SmelC593 transcript (putative cis-encoded mRNA leader)

**>SmelA080**

1304774_1305065 (SMa2335_SMa2337)


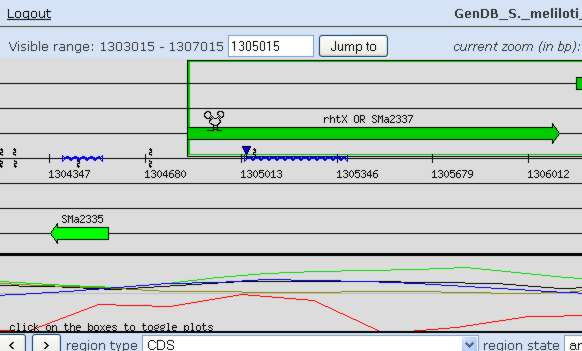
GATCTTCGACGCTGCGCCGGAAGCGTCGGCTGATCTTTCTCTCTCTCTCTCTCTCTCTCTCTCTCTCTCTCTCTCTCTCCTCGCTCGAGTAAGAGCTGTTGCAGCAGAGAATGCGCCCTGGAGGCGTCCTATCGCCTCTCTCGAAAATGCGTTCGCTACTGTCTTAATGAGGTTCGCTCACATCCAAGCCGTTCACCGCACGTCCATTTAAAGATGACGGCAACACTCATGTTTATCGTCAGACAATGTTGCCGGGCAGTGGCAGTTTTCGATGCTCGCCGCTGTGGTCCAAGGATCGGATCCTATCATGACAATTGCGCAGACTTCTCCAGCGGTCCGCGAAGGGTCGACGGCGGCAGGTGCGGGAAGGCTTTACGCGGTCTTGGGCGGACTCTACCTCGCCCAGGGCATCCCAACCTACCTCCTGCTCGTGGCCCTGCCGCCCTTGATGCGCGAGAGCGGTGCATCGCGCACCGCGATCGGCCTGTTCTCGCTTCTGATGTTGCCGCTAGTGCTGAAATTCGCGGTTGCGCCTCTCGTTGATCGTT…

RirA box

SmelA080 transcript (putative cis-encoded mRNA leader)

rhtX ORF


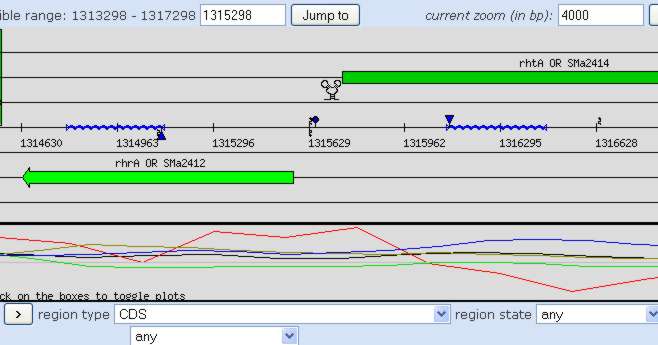
**>SmelA091**

1315674_1315741 (SMa2412_SMa2414)

GTCGTGCGCCAGCCTTTCCTGTTGACGTTCGCATGCGTCCAAATGAGGTTCGCCATTATCCAAGCGGCGAACACCCTTAGCCCATAAAACATGACTTAAATAGTCTTGTATTGGCAATTTGCCCGCCCACCGGCAGCGGCAATTGTTTTCTGGTGCGCAGGGGGCGTTATGGGCAACAATGAGAATGGCGGCATAAGCTTTTGCGTCTTCGTCGTCGTAATTGGATTTGGCACGGGTGCTGTTGCGCAGGAGCCAGCGAATCAATCCGAAGCTG

RirA box

SmelA091 transcript (putative cis-encoded mRNA leader)

rhtA ORF

The three loci contain RirA boxes possibly overlapping the promoter of genes or operons that are controlled by iron availability and RirA [11,15]. The identified small transcripts could be derived from processing of the mRNA leaders. Thus, we carried out target mRNA prediction using TargetRNA, as described above, to look for potential genes related to iron metabolism that could be targeted by the presumed RirA-dependent sRNAs. The prediction results are summarized in Table S3. Overall, no evident genes or operons directly related to iron usage or metabolism (i.e., bacterioferritin, siderophore synthesis, iron transport, cytochromes) were detected among the hits with higher scores for the three queried sRNA transcripts.

c. Is the *bfr* mRNA targeted by any putative RirA-dependent sRNA? Finally, as bacterioferritin was one of the iron related proteins that appeared upregulated in the *hfq* mutant, we tried to identify IgRs which may potentially encode sRNAs under the control of the iron regulator RirA, by carrying out reverse TargetRNA search on the chromosome of *S. meliloti* 1021, using the *bfr* leader region (±30 bases around the start codon, with a seed region of 7 bases and allowing G:U pairs). The results are presented in Table S3. Only one among the eleven predicted IgRs did contain an annotated sRNA transcript, but there was no putative RirA binding sequence co-localized with the transcript.

***Conclusions***

As there is no experimental evidence for the expression of RyhB-like sRNAs in *S. meliloti*, we scanned IgRs for the presence of putative RirA binding sites located upstream annotated non-coding RNAs, following a similar strategy to that reported for identification of the *P. aeruginosa* Fur-regulated PrrF1 and PrrF2 sRNAs [9]. The search identified three annotated small transcripts just downstream putative RirA binding sites. All four hits corresponded to transcripts detected by RNA pyrosequencing as cis-regulatory mRNA leaders, with three of them linked to iron-metabolism genes. Target mRNA searches for these annotated non-coding transcripts did not reveal obvious iron-related mRNAs. Furthermore, reverse TargetRNA search of putative sRNA regulators of the bacterioferritin mRNA did neither identify possible RNA genes under the control of RirA. Thus, the identification of bona-fide RyhB homolog(s) in *S. meliloti* remains an open task.

**References**

1. Sharma CM, Darfeuille F, Plantinga TH, Vogel J (2007) A small RNA regulates multiple ABC transporter mRNAs by targeting C/A-rich elements inside and upstream of ribosome-binding sites. Genes Dev 21: 2804-2817.

2. Urbanowski ML, Stauffer LT, Stauffer GV (2000) The gcvB gene encodes a small untranslated RNA involved in expression of the dipeptide and oligopeptide transport systems in Escherichia coli. Mol Microbiol 37: 856-868.

3. Caswell CC, Gaines JM, Ciborowski P, Smith D, Borchers CH, et al. (2012) Identification of two small regulatory RNAs linked to virulence in Brucella abortus 2308. Mol Microbiol 85: 345-360.

4. Wilms I, Voss B, Hess WR, Leichert LI, Narberhaus F (2011) Small RNA-mediated control of the Agrobacterium tumefaciens GABA binding protein. Mol Microbiol 80: 492-506.

5. del Val C, Romero-Zaliz R, Torres-Quesada O, Peregrina A, Toro N, et al. (2012) A survey of sRNA families in alpha-proteobacteria. RNA Biol 9: 119-129.

6. Torres-Quesada O, Oruezabal RI, Peregrina A, Jofre E, Lloret J, et al. (2010) The Sinorhizobium meliloti RNA chaperone Hfq influences central carbon metabolism and the symbiotic interaction with alfalfa. BMC Microbiol 10: 71.

7. Balbontin R, Fiorini F, Figueroa-Bossi N, Casadesus J, Bossi L (2010) Recognition of heptameric seed sequence underlies multi-target regulation by RybB small RNA in Salmonella enterica. Mol Microbiol 78: 380-394.

8. Bouvier M, Sharma CM, Mika F, Nierhaus KH, Vogel J (2008) Small RNA binding to 5' mRNA coding region inhibits translational initiation. Mol Cell 32: 827-837.

9. Salvail H, Masse E (2012) Regulating iron storage and metabolism with RNA: an overview of posttranscriptional controls of intracellular iron homeostasis. Wiley Interdiscip Rev RNA 3: 26-36.

10. Platero R, Peixoto L, O'Brian MR, Fabiano E (2004) Fur is involved in manganese-dependent regulation of mntA (sitA) expression in Sinorhizobium meliloti. Appl Environ Microbiol 70: 4349-4355.

11. Chao TC, Buhrmester J, Hansmeier N, Puhler A, Weidner S (2005) Role of the regulatory gene rirA in the transcriptional response of Sinorhizobium meliloti to iron limitation. Appl Environ Microbiol 71: 5969-5982.

12. Rodionov DA, Gelfand MS, Todd JD, Curson AR, Johnston AW (2006) Computational reconstruction of iron- and manganese-responsive transcriptional networks in alpha-proteobacteria. PLoS Comput Biol 2: e163.

13. Schluter JP, Reinkensmeier J, Daschkey S, Evguenieva-Hackenberg E, Janssen S, et al. (2010) A genome-wide survey of sRNAs in the symbiotic nitrogen-fixing alpha-proteobacterium Sinorhizobium meliloti. BMC Genomics 11: 245.

14. Valverde C, Livny J, Schluter JP, Reinkensmeier J, Becker A, et al. (2008) Prediction of Sinorhizobium meliloti sRNA genes and experimental detection in strain 2011. BMC Genomics 9: 416.

15. Amarelle V, Koziol U, Rosconi F, Noya F, O'Brian MR, et al. (2010) A new small regulatory protein, HmuP, modulates haemin acquisition in Sinorhizobium meliloti. Microbiology 156: 1873-1882.
